# Draft Genome Sequence of *Pseudomonas* sp. Strain 2-92, a Biological Control Strain Isolated from a Field Plot Under Long-Term Mineral Fertilization

**DOI:** 10.1128/genomeA.01121-13

**Published:** 2014-01-09

**Authors:** Zaky Adam, James Tabi Tambong, Qing Chen, Christopher T. Lewis, C. André Lévesque, Renlin Xu

**Affiliations:** Biodiversity, Environmental Health Program, Agriculture and Agri-Food Canada, Ottawa, Ontario, Canada

## Abstract

*Pseudomonas* sp. strain 2-92, isolated from a Canadian field plot under long-term mineral fertilization, strongly inhibits the growth of *Fusarium graminearum*, *Rhizoctonia solani*, and *Gaeumannomyces graminis*. Here, we report the draft genome sequence of *Pseudomonas* sp. strain 2-92.

## GENOME ANNOUNCEMENT

*Pseudomonas* sp. strain 2-92, isolated from a Canadian field plot under long-term (>40 years) mineral fertilization, is a potent biological control agent against *Fusarium graminearum*, *Rhizoctonia solani*, and *Gaeumannomyces graminis*. Here, we report the draft genome sequence of *Pseudomonas* sp. 2-92. The whole-genome sequence was determined by paired-end sequencing using an Illumina HiSeq instrument with TrueSeq V3 chemistry (Génome-Québec, Montreal, Canada). A total of 11,208,890 paired-end reads, each 150 bp in length, were generated from inserts of 395 bp. Quality checking using FastQC (http://www.bioinformatics.babraham.ac.uk/projects/fastqc/) showed that the reads were of sufficiently good quality that there was no need for any trimming or error correction. Initial *de novo* assembly, using ABySS (1), produced 31 contigs contained in 27 scaffolds, among which scaffolds with lengths of <400 bp (2) were removed. The remaining 23 scaffolds (minimum, 2,094 bp; maximum, 1,349,360 bp; N_50_, 622,277 bp; total size, 6,418,883 bp; total number of Ns, 1,603) were used for further analyses. SSPACE (3) was then run on the resulting scaffolds to extend and merge them into larger scaffolds based on read-pair information and short overlaps, reducing the number of scaffolds to 19 (minimum, 2,112 bp; maximum, 1,349,360 bp; N_50_, 622,357 bp; total size, 6,419,850 bp; total number of Ns, 1,607). GapFiller (2) was then run in order to close the gaps between the short scaffolds that are contained within the 19 large scaffolds by replacing the unknown nucleotide Ns with true nucleotides based on read-pair information and short overlaps. This resulted in a final draft assembly of 6,419,614 bp containing 29 Ns and consisting of 19 scaffolds (minimum, 2,112 bp; maximum, 1,349,399 bp; N_50_, 622,148 bp), with a G+C content 60.40% and an overall coverage estimate of 261×.

Mauve Contig Mover (4) was used to order the *Pseudomonas* sp. 2-92 scaffolds relative to *Pseudomonas fluorescens* SBW25 (5) (GenBank accession no. NC_012660.1). *P. fluorescens* SBW25 has been extensively studied for its ability to protect peas from seedling damping-off caused by the oomycete *Pythium ultimatum* (6). Automated annotation was performed using the RAST annotation server (7). *Pseudomonas* sp. 2-92 contains 5,836 predicted protein-encoding sequences, of which 5,018 are assigned functions, 370 have proposed functions, and 448 are considered to encode hypothetical proteins. Also, *Pseudomonas* sp. 2-92 contains 88 predicted noncoding RNAs, of which 69 are tRNAs and 19 are rRNAs. The numbers of copies of 16S rRNA, 5S rRNA, and 23S rRNA are 7, 7, and 5, respectively. Running Glimmer (8) to predict genes based on open reading frames (ORFs) as a training set resulted in 5,842 genes, and the results of running RNAmmer (9) and tRNAscan-SE (10) to predict rRNA genes and tRNA genes, respectively, were identical to those of RAST. A detailed comparative genomic analysis of the draft genome sequence of *Pseudomonas* sp. 2-92 and those of well-documented biocontrol *P. fluorescens* strains will follow in a subsequent publication.

### Nucleotide sequence accession numbers.

This whole-genome shotgun project has been deposited at DDBJ/EMBL/GenBank under the accession no. AYTD00000000. The version described in this paper is version AYTD01000000.
